# Endometriosis Uncovered: From Chronic Inflammation to Reproductive Dysfunction and Impaired ART Outcomes

**DOI:** 10.3390/medicina62050885

**Published:** 2026-05-05

**Authors:** Luana Ghilea Seleș, Viorela Romina Murvai, Laura Maghiar, Alin Bodog, Anca Huniadi

**Affiliations:** 1 Doctoral School of Biological and Biomedical Sciences, University of Oradea, 1 University Street, 410087 Oradea, Romania; 2 Calla-Infertility Diagnostic and Treatment Center, Constantin A. Rosetti Street, 410103 Oradea, Romania; dr.rominamurvai@gmail.com; 3 Department of Psychoneurosciences and Rehabilitation, University of Oradea, 1 University Street, 410087 Oradea, Romania; laura.maghiar@uoradea.ro; 4 Department of Surgical Disciplines, Faculty of Medicine and Pharmacy, University of Oradea, 10 Piata 1 Decembrie Street, 410073 Oradea, Romania; alin.bodog@didactic.uoradea.ro (A.B.); ahuniadi@uoradea.ro (A.H.)

**Keywords:** endometriosis, inflammation, immune dysregulation, infertility, cytokines, assisted reproductive technology, biomarkers

## Abstract

*Background and Objectives*: Endometriosis is a chronic, estrogen-dependent disorder characterized by the presence of functional endometrial tissue, comprising both glandular and stromal components, located outside the uterine cavity, affecting approximately 6–10% of women of reproductive age and up to 30–50% of those with infertility. Increasing evidence indicates that endometriosis is not solely a localized gynecological condition but rather a systemic inflammatory and immune-mediated disease. Chronic inflammation and immune dysregulation contribute to disease progression and may impair reproductive function. This review aims to analyze the current evidence regarding the inflammatory and immunological mechanisms underlying endometriosis and to evaluate their impact on reproductive dysfunction and assisted reproductive technology (ART) outcomes. *Materials and Methods*: A comprehensive narrative review was conducted using major scientific databases, including PubMed, Scopus, and Web of Science. Relevant articles published in the last decade were selected using keywords such as “endometriosis”, “inflammation”, “immune response”, “cytokines”, and “ART outcomes”. Both clinical and experimental studies were included to assess the relationship between inflammatory markers, immune alterations, and reproductive performance. *Results*: Endometriosis is associated with elevated levels of pro-inflammatory cytokines, including interleukin (IL)-1β, IL-6, and tumor necrosis factor-alpha (TNF-α), as well as increased oxidative stress and altered peritoneal microenvironment. Immune dysfunction is characterized by activated macrophages, decreased natural killer (NK) cell cytotoxicity, and an imbalance in T-cell populations. These alterations negatively affect oocyte quality, fertilization, embryo development, and endometrial receptivity. Emerging biomarkers such as IL-6, TNF-α, and CA-125 show potential in predicting disease severity and ART outcomes, although their clinical utility remains under investigation. *Conclusions*: Endometriosis should be regarded as a systemic immuno-inflammatory disorder with significant implications for reproductive health. The interaction between chronic inflammation and immune dysregulation plays a central role in infertility and suboptimal ART outcomes. Further research is required to validate reliable biomarkers and develop targeted therapeutic strategies to improve reproductive success in affected patients.

## 1. Introduction

Despite significant advances in gynecological research and assisted reproductive technologies (ARTs), endometriosis remains one of the most complex and poorly understood disorders affecting women of reproductive age. It is defined by the presence of functional endometrial tissue, including both glandular and stromal components, located outside the uterine cavity, leading to a chronic inflammatory condition associated with pelvic pain, infertility, and reduced quality of life [1,2]. Epidemiological data indicate that endometriosis affects approximately 6–10% of women of reproductive age, with prevalence rising to 30–50% among infertile patients, highlighting its substantial reproductive impact [3,4].

Traditionally, endometriosis has been considered a localized pelvic disease, primarily explained by retrograde menstruation and implantation of endometrial cells into the peritoneal cavity [5]. However, this theory alone fails to explain disease occurrence at distant anatomical sites, the variability in clinical presentation, and the persistence of lesions despite treatment [6]. Increasing evidence suggests that endometriosis should be redefined as a systemic disorder involving complex interactions between hormonal, inflammatory, and immune pathways [7,8].

Chronic inflammation represents a central feature of endometriosis, characterized by elevated levels of pro-inflammatory cytokines such as interleukin (IL)-1β, IL-6 and tumor necrosis factor-alpha (TNF-α), as well as increased oxidative stress within the peritoneal microenvironment [9,10,11]. These alterations promote angiogenesis, cellular proliferation, and lesion survival. In parallel, immune dysregulation plays a crucial role in disease progression, including macrophage activation, reduced natural killer (NK) cell cytotoxicity, and imbalances in T-cell subpopulations [12,13,14]. This impaired immune surveillance allows ectopic endometrial cells to evade clearance and establish persistent lesions.

The reproductive consequences of these immuno-inflammatory alterations are increasingly recognized. The altered peritoneal and follicular environments negatively affect oocyte quality, fertilization, embryo development, and endometrial receptivity [15,16,17]. Emerging evidence suggests that chronic inflammation directly interferes with key reproductive processes, including folliculogenesis and oocyte maturation, thereby reducing developmental competence at early embryonic stages. As a result, women with endometriosis often experience reduced success rates in ART procedures, including in vitro fertilization (IVF) and intracytoplasmic sperm injection (ICSI), as well as higher rates of implantation failure and early pregnancy loss [18,19]. From a clinical perspective, these alterations may contribute to impaired implantation dynamics and increased risk of early pregnancy loss, reinforcing the multifactorial nature of endometriosis-associated infertility. Nevertheless, the extent to which inflammatory and immune mechanisms directly influence ART outcomes remains a subject of ongoing debate, with some studies reporting significant associations, while others suggest more modest or indirect effects [20,21].

In recent years, considerable attention has been directed toward identifying reliable biomarkers that reflect disease activity and predict reproductive outcomes. Molecules such as IL-6, TNF-α, and CA-125, along with emerging candidates including microRNAs and extracellular vesicles, have shown potential in improving diagnosis and guiding therapeutic strategies [22,23,24]. However, their clinical applicability is still limited by variability in study design, lack of standardization, and insufficient validation in large cohorts.

Given these complexities, there is a growing need for an integrative understanding of endometriosis that bridges molecular mechanisms with clinical outcomes. This narrative review aims to synthesize current evidence on the inflammatory and immunological pathways involved in endometriosis and to elucidate their impact on reproductive dysfunction and ART outcomes. By integrating mechanistic insights with clinical data, we propose a conceptual framework linking chronic inflammation, immune dysregulation, and impaired reproductive performance, highlighting potential avenues for personalized therapeutic strategies and improved patient management.

## 2. Materials and Methods

### 2.1. Study Design

This study was conducted as a comprehensive narrative review aimed at synthesizing current evidence regarding the inflammatory and immunological mechanisms underlying endometriosis and their impact on reproductive dysfunction and assisted reproductive technology (ART) outcomes. The review was designed to integrate findings from both basic science and clinical research in order to provide a translational perspective on disease pathophysiology and reproductive implications.

### 2.2. Data Sources and Search Strategy

A comprehensive literature search was conducted using major electronic databases, including PubMed/MEDLINE, Scopus, and Web of Science. The search included studies published between 2015 and 2026 and was last updated in January 2026. This time frame was selected to ensure the inclusion of recent, clinically relevant evidence reflecting current advances in endometriosis and reproductive medicine.

The search strategy was designed to ensure broad coverage of the topic while maintaining the flexibility appropriate for a narrative review. Study identification and selection were performed using predefined keywords and eligibility criteria. The following keywords and Boolean combinations were applied: “endometriosis” AND (“inflammation” OR “immune response” OR “cytokines” OR “oxidative stress” OR “ART outcomes” OR “IVF” OR “infertility” OR “biomarkers”). The search strategy was adapted for each database to maximize sensitivity and relevance. The search terms were applied in titles, abstracts, and keywords to ensure comprehensive retrieval of relevant studies.

Filters were applied to include original research articles, clinical studies, and systematic reviews, while excluding case reports, editorials, and conference abstracts lacking full data. Only articles published in English were considered.

Additional relevant studies were identified through manual screening of the reference lists of selected articles to ensure comprehensive coverage of the topic.

The initial search yielded approximately 80 articles. After screening for relevance and applying the predefined eligibility criteria, approximately 52 studies were included in the final analysis. The selection process aimed to ensure the inclusion of the most relevant and up-to-date evidence, with a particular focus on studies that provide mechanistic insights and clinically relevant data on reproductive outcomes and assisted reproductive technologies.

### 2.3. Eligibility Criteria

Studies were selected based on predefined inclusion criteria. Eligible publications included original research articles, clinical studies, and systematic reviews that investigated the inflammatory, immunological, or molecular mechanisms involved in endometriosis. Particular emphasis was placed on studies evaluating reproductive outcomes, including natural fertility as well as assisted reproductive technologies (ARTs), such as in vitro fertilization (IVF) and intracytoplasmic sperm injection (ICSI). Only articles published in English were considered for inclusion.

Studies were excluded if they consisted of case reports, editorials, or conference abstracts lacking full data. Additionally, publications lacking a clear methodological description, or those not directly addressing reproductive outcomes or immune–inflammatory mechanisms in endometriosis, were not considered eligible for analysis.

### 2.4. Study Selection and Data Extraction

The selection process involved initial screening of titles and abstracts, followed by full-text evaluation of potentially relevant studies. Data were extracted and organized according to key domains, including inflammatory mediators, immune cell alterations, reproductive outcomes, and ART performance indicators.

Special emphasis was placed on identifying mechanistic links between immune–inflammatory pathways and clinical reproductive outcomes.

The study selection process followed a stepwise approach, including identification, screening, eligibility assessment, and final inclusion of relevant studies.

### 2.5. Data Synthesis and Analysis

Given the heterogeneity in study designs, patient populations, and outcome measures, a qualitative synthesis approach was employed. The selected studies were comparatively analyzed to identify consistent patterns, areas of divergence, and emerging trends within the current body of literature.

The synthesized findings were subsequently organized into thematic sections, addressing key aspects of the disease, including inflammatory pathways, immune dysregulation, reproductive dysfunction, outcomes related to assisted reproductive technologies (ARTs), and the potential role of biomarkers in diagnosis and prognosis.

This study was conducted as a narrative review using a structured and transparent approach to literature identification and selection. However, it does not follow a formal systematic review protocol and should not be interpreted as one. A formal PRISMA flow diagram was not included because the objective of this work was to provide a qualitative, integrative synthesis of the available evidence rather than a quantitative systematic analysis.

## 3. Results

### 3.1. Pro-Inflammatory Microenvironment

Endometriosis is characterized by the establishment of a persistent pro-inflammatory microenvironment, particularly within the peritoneal cavity, which plays a central role in disease initiation and progression Table 1. This altered microenvironment is driven by increased concentrations of pro-inflammatory cytokines, chemokines, and growth factors that collectively promote lesion survival, angiogenesis, and tissue remodeling [1,4,25].

**Table 1:** Major inflammatory mediators involved in endometriosis and their biological effects.

Mediator	Source	Main Effects	Relevance in Endometriosis	References
IL-1β	Macrophages	Pro-inflammatory signaling, cell proliferation	Promotes lesion establishment	[3,4,15]
IL-6	Immune cells, endometrial cells	Angiogenesis, immune modulation	Associated with disease severity	[3,4,13,15]
TNF-α	Macrophages	Chronic inflammation, apoptosis resistance	Supports lesion survival	[3,4,15]
PGE2 (prostaglandin E2)	Endometrial cells	Estrogen production, inflammation amplification	Drives hormonal–inflammatory loop	[5,13,17]
ROS (reactive oxygen species)	Multiple sources	Oxidative stress, DNA damage	Impairs oocyte quality	[4,5,19]

Among the most extensively studied mediators are interleukin (IL)-1β, IL-6, and tumor necrosis factor-alpha (TNF-α), which are consistently elevated in the peritoneal fluid and serum of patients with endometriosis. These cytokines contribute to the activation of inflammatory signaling pathways, enhance cellular proliferation, and stimulate the expression of adhesion molecules, facilitating the implantation and persistence of ectopic endometrial tissue [3,4,26]. In addition, IL-6 has been implicated in promoting angiogenesis and immune modulation, while TNF-α plays a key role in sustaining chronic inflammation and inducing apoptotic resistance in ectopic cells.

These findings are supported by multiple recent studies reporting similar inflammatory alterations in endometriosis [3,4,13].

Prostaglandins, particularly prostaglandin E2 (PGE2), further amplify the inflammatory cascade by stimulating local estrogen production through aromatase activation, thereby creating a self-perpetuating feedback loop between inflammation and hormonal dysregulation [5,27]. This interaction contributes to the estrogen-dependent nature of the disease and supports the growth and maintenance of endometriotic lesions.

Oxidative stress is another critical component of the pro-inflammatory microenvironment. Elevated levels of reactive oxygen species (ROS) and reduced antioxidant capacity have been documented in the peritoneal fluid of affected patients, leading to cellular damage, lipid peroxidation, and DNA instability [4,28]. These alterations not only exacerbate inflammation but also impair cellular function, particularly within the ovarian and peritoneal compartments.

Importantly, this pro-inflammatory milieu extends beyond local tissue effects and has significant implications for reproductive function. The altered biochemical environment negatively influences oocyte quality, disrupts folliculogenesis, and compromises the developmental competence of embryos. Furthermore, the inflammatory state impairs endometrial receptivity, thereby reducing the likelihood of successful implantation [5,29,30].

Collectively, the pro-inflammatory microenvironment represents a fundamental driver of endometriosis pathophysiology, acting as a key link between molecular alterations and clinical manifestations, including infertility and suboptimal outcomes in assisted reproductive technologies.

### 3.2. Immune Dysregulation

Beyond the pro-inflammatory microenvironment, endometriosis is characterized by profound alterations in immune function, which contribute to the persistence and progression of ectopic lesions. Under physiological conditions, immune surveillance mechanisms identify and eliminate displaced endometrial cells. However, in endometriosis, these mechanisms are impaired, allowing ectopic tissue to evade immune clearance and establish a chronic pathological state [2,31].

Macrophages are among the most prominent immune cell populations involved in endometriosis. Increased numbers of activated peritoneal macrophages have been consistently reported in affected patients. These cells exhibit an altered functional phenotype, characterized by enhanced secretion of pro-inflammatory cytokines, growth factors, and angiogenic mediators [4,5,32,33,34]. Rather than facilitating tissue clearance, macrophages in endometriosis contribute to lesion maintenance, neovascularisation, and fibrosis, thereby reinforcing the inflammatory microenvironment. The main immune cell alterations involved in endometriosis are summarized in Table 2.

Natural killer (NK) cells, which play a crucial role in immune surveillance, also exhibit functional impairment in endometriosis. A reduction in NK cell cytotoxicity has been widely documented, limiting their ability to eliminate ectopic endometrial cells [2,3]. This diminished activity is thought to result from both intrinsic cellular dysfunction and the inhibitory effects of the surrounding inflammatory milieu. Consequently, ectopic cells can survive and proliferate within the peritoneal cavity.

T-cell dysregulation further contributes to immune imbalance in endometriosis. Alterations in T-helper cell subsets, including a shift in the Th1/Th2 balance and an increase in regulatory T cells (Treg), promote an immunotolerant environment that favors lesion persistence [4]. This imbalance reduces effective immune responses while enhancing mechanisms that locally suppress inflammation, paradoxically allowing chronic disease to persist.

These findings are supported by multiple recent studies reporting similar immune alterations in endometriosis [4,5,14].

In addition, emerging evidence suggests that endometriosis shares features with autoimmune disorders, including the presence of autoantibodies and aberrant immune activation [5]. These findings support the concept that endometriosis is not solely a localized condition but rather a systemic immunological disorder with complex regulatory disturbances.

Emerging evidence has documented the presence of several autoantibodies in women with endometriosis, including anti-endometrial, antiphospholipid, and antinuclear antibodies, although their exact pathogenic role remains poorly defined [5,14].

Importantly, immune dysregulation does not act in isolation but interacts closely with inflammatory pathways, forming a self-perpetuating cycle that exacerbates disease progression. This interplay contributes to the establishment of a permissive microenvironment that negatively affects reproductive function, further linking immune alterations to infertility and impaired outcomes in assisted reproductive technologies.

### 3.3. Oxidative Stress and Cellular Damage

Oxidative stress represents a key component of endometriosis pathophysiology and acts as a critical link between inflammation, immune dysregulation, and impaired reproductive function. It is characterized by an imbalance between the production of reactive oxygen species (ROS) and the antioxidant defense mechanisms, leading to cellular and molecular damage within the peritoneal and ovarian environments [4,5].

In patients with endometriosis, elevated levels of ROS have been consistently detected in the peritoneal fluid, follicular fluid, and systemic circulation. These reactive species are primarily generated by activated immune cells, particularly macrophages, and by ectopic endometrial tissue itself [3,4]. The sustained production of ROS contributes to lipid peroxidation, protein modification, and DNA damage, ultimately disrupting normal cellular function and promoting disease progression.

Oxidative stress also plays a pivotal role in modulating key reproductive processes. Within the ovarian microenvironment, excessive ROS levels impair oocyte quality by affecting mitochondrial function, reducing ATP production, and inducing apoptotic pathways. These alterations compromise oocyte maturation and fertilization potential, thereby negatively influencing reproductive outcomes [3,5].

In addition to female-related factors, growing evidence suggests that oxidative stress may also adversely affect sperm parameters, particularly by increasing sperm DNA fragmentation (SDF). Elevated levels of reactive oxygen species (ROS) are associated with reduced sperm quality, impaired fertilization capacity, and altered embryo development. Clinically, higher SDF levels have been correlated with lower fertilization rates, decreased embryo quality, and increased risk of implantation failure and early pregnancy loss [3,19,20].

Furthermore, oxidative damage extends to the endometrium, where it alters cellular signaling and reduces endometrial receptivity. Changes in gene expression, including those related to implantation markers and adhesion molecules, contribute to implantation failure and early pregnancy loss. In addition, ROS may interfere with embryonic development by affecting cellular integrity and division.

Importantly, oxidative stress interacts synergistically with inflammatory and immune pathways, forming a self-amplifying cycle that exacerbates tissue damage and disease persistence. This interconnected network further highlights the multifactorial nature of endometriosis and its impact on fertility.

The major sources and biological effects of oxidative stress in endometriosis are summarized in Table 3.

### 3.4. The Inflammation–Immune–Oxidative Axis

Endometriosis is increasingly recognized as a multifactorial disorder driven by the intricate interplay between chronic inflammation, immune dysregulation, and oxidative stress. These interconnected mechanisms do not act independently but rather form a dynamic and self-perpetuating network that underlies both disease progression and reproductive impairment.

**Figure 1:**
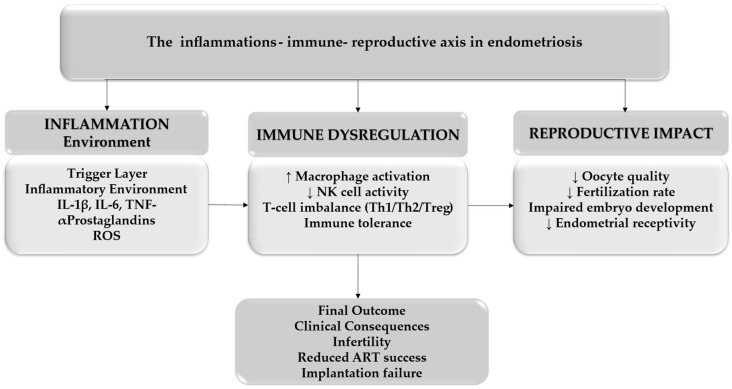
Inflammation–immune–reproductive axis in endometriosis. Arrows between boxes indicate the direction of interaction between biological processes. Upward arrows (↑) denote increased activity or expression, while downward arrows (↓) indicate decreased activity or suppression of the analyzed parameters.

As illustrated in Figure 1, the establishment of a pro-inflammatory microenvironment represents the initial trigger in this cascade. Elevated levels of cytokines, including IL-1β, IL-6, and TNF-α, promote the activation of immune cells and enhance the production of reactive oxygen species (ROS). This inflammatory milieu does not represent a strictly linear process but rather a bidirectional interaction. Initial immune dysfunction may impair the clearance of refluxed endometrial cells, facilitating their implantation, while the resulting ectopic lesions further amplify local inflammation and immune dysregulation, creating a self-perpetuating cycle [3,14]. Immune dysregulation further amplifies this process by impairing the clearance of ectopic tissue. Activated macrophages, reduced natural killer (NK) cell cytotoxicity, and alterations in T-cell subsets contribute to an immunotolerant environment that allows lesion persistence. In parallel, oxidative stress exacerbates cellular damage through lipid peroxidation, mitochondrial dysfunction, and DNA instability, thereby reinforcing both inflammatory and immune pathways.

The cumulative effect of these alterations is the development of a hostile reproductive microenvironment. At the ovarian level, oxidative and inflammatory stress impair oocyte quality and maturation, whereas at the endometrial level, they disrupt receptivity and implantation. Embryo development is also negatively affected, leading to reduced fertilization rates and compromised embryo viability [35].

Importantly, these mechanisms establish a vicious cycle in which inflammation, immune dysfunction, and oxidative stress continuously reinforce one another, contributing to the maintenance of chronic disease. This integrated model provides a mechanistic explanation for the reduced success rates observed in assisted reproductive technologies (ARTs) among patients with endometriosis, including lower implantation rates and increased risk of early pregnancy loss.

**Figure 2:**
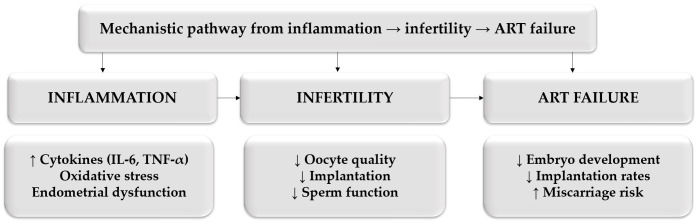
Mechanistic pathway linking inflammation, infertility, and ART failure in endometriosis. Upward arrows (↑) indicate increased activity, while downward arrows (↓) indicate decreased activity. Arrows between boxes represent directional interactions.

Understanding this complex interplay is essential for developing targeted therapeutic strategies. By addressing not only the anatomical manifestations of the disease but also its underlying molecular and immunological drivers, it may be possible to improve reproductive outcomes and optimize individualized treatment approaches. The integrated mechanistic pathway linking inflammation, immune dysregulation, oxidative stress, and reproductive impairment in endometriosis is illustrated in Figure 2.

### 3.5. Biomarkers and Clinical Implications

In addition to mechanistic insights, increasing attention has been directed toward identifying reliable biomarkers that reflect the inflammatory, immune, and oxidative processes underlying endometriosis. These biomarkers have the potential not only to improve diagnostic accuracy but also to provide valuable information regarding disease severity and reproductive prognosis, particularly in the context of assisted reproductive technologies (ARTs) [1,3,36].

From a clinical perspective, although several inflammatory and immune biomarkers such as IL-6, TNF-α, and CA-125 have been extensively investigated, their routine use in diagnosis remains limited. CA-125 is currently the most widely used biomarker in clinical practice; however, it lacks sufficient specificity and sensitivity, particularly in early-stage disease [5,20]. Similarly, cytokine-based markers demonstrate variability across studies, limiting their standalone diagnostic value [3,4]. As a result, these biomarkers are more commonly used as adjunctive tools rather than definitive diagnostic indicators.

Among the most extensively investigated biomarkers are pro-inflammatory cytokines, including interleukin (IL)-6, tumor necrosis factor-alpha (TNF-α), and IL-1β, which are consistently elevated in the serum, peritoneal fluid, and follicular environment of patients with endometriosis [3,4,13]. These molecules play a central role in sustaining chronic inflammation and have been associated with impaired oocyte quality, altered folliculogenesis, and reduced embryo developmental potential [19,20]. In addition, elevated levels of prostaglandins and oxidative stress markers further contribute to the disruption of the reproductive microenvironment, negatively affecting fertilization and implantation processes [4,5].

CA-125 remains one of the most widely used clinical biomarkers in endometriosis, particularly in moderate-to-severe disease stages. Although its specificity is limited, it may provide supportive information when combined with imaging and clinical findings [5]. More recently, emerging biomarkers such as microRNAs and extracellular vesicles have attracted interest for their potential roles in regulating gene expression and intercellular communication within the endometrial and immune systems [3,16,25].

From a reproductive perspective, the clinical applicability of these biomarkers is increasingly relevant. Alterations in inflammatory and oxidative profiles have been correlated with reduced ovarian responsiveness, decreased oocyte competence, and suboptimal ART outcomes, including lower implantation and live birth rates [26,35,37]. However, the predictive value of individual biomarkers remains inconsistent, largely due to heterogeneity in study populations, disease phenotypes, and methodological approaches [20,38,39,40].

Consequently, current research is shifting toward integrative approaches that combine multiple biomarkers into composite panels, aiming to improve patient stratification and personalize therapeutic strategies. Such approaches may enable clinicians to optimize ovarian stimulation protocols, select appropriate timing for ART interventions, and monitor treatment response more effectively [14,41].

In addition to diagnostic implications, the inflammatory and immune pathways described in endometriosis also represent potential therapeutic targets. Strategies aimed at modulating inflammatory cytokines, reducing oxidative stress, or altering immune responses have been explored, including GnRH analogues, anti-inflammatory agents, and emerging immunomodulatory therapies [38,39,40]. However, the clinical effectiveness of these approaches varies, and current treatments primarily focus on hormonal suppression rather than direct targeting of immune or inflammatory pathways [18,39].

**Figure 3:**
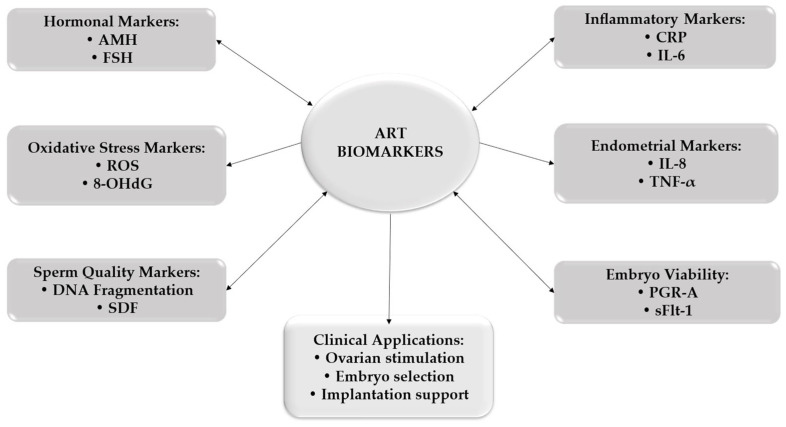
Integration of biomarkers in assisted reproductive technologies (ARTs). AMH—anti-Müllerian hormone; FSH—follicle-stimulating hormone; ROS—reactive oxygen species; 8-OHdG—8-hydroxy-2′-deoxyguanosine; DNA—deoxyribonucleic acid; SDF—sperm DNA fragmentation; CRP—C-reactive protein; IL-6—interleukin-6; IL-8—interleukin-8; TNF-α—tumor necrosis factor-alpha; PGR-A—progesterone receptor A; sFlt-1—soluble fms-like tyrosine kinase-1.

The potential clinical integration of inflammatory and immune biomarkers in predicting ART outcomes is summarized in Figure 3.

## 4. Discussion

The present review highlights the complex interplay between chronic inflammation, immune dysregulation, and oxidative stress in the pathogenesis of endometriosis and their cumulative impact on reproductive outcomes. Beyond being a localized gynecological condition, endometriosis is increasingly recognized as a systemic disorder with significant implications for fertility, quality of life, and long-term health trajectories [24,25,26].

The current literature presents notable heterogeneity regarding the impact of endometriosis on ART outcomes. While several studies report reduced ovarian responsiveness, impaired embryo development, and lower cumulative live birth rates, other investigations suggest that certain outcomes, particularly implantation rates, may not be consistently affected. These discrepancies may be explained by differences in study design, patient selection, disease severity, and ART protocols, highlighting the complexity of establishing direct causal relationships [27,28,29].

From a reproductive perspective, one of the most clinically relevant consequences of endometriosis is its negative influence on assisted reproductive technology (ART) outcomes. Several recent systematic reviews and meta-analyses have demonstrated that endometriosis is associated with reduced cumulative live birth rates, altered ovarian response, and impaired embryo availability [26,30,31,32]. However, the extent of this impact remains variable. While some studies report significant impairments across multiple reproductive endpoints, others suggest that implantation rates may not always be consistently affected [33]. This variability highlights the complexity of the disease and suggests that reproductive impairment is not uniform across all patient populations.

The effect of endometriosis on IVF outcomes appears to be multifactorial, involving both local and systemic mechanisms. Alterations in the follicular microenvironment, including increased oxidative stress and elevated inflammatory mediators, contribute to decreased oocyte quality and impaired granulosa cell function, ultimately affecting embryo development [19]. In addition, ovarian endometriomas have been associated with reduced ovarian responsiveness, although their direct impact on pregnancy rates remains debated [32,34,35]. These inconsistencies across studies may be explained by differences in disease severity, patient selection, and ART protocols.

Another key aspect is the role of endometrial receptivity. Even when oocyte quality is relatively preserved, alterations in the eutopic endometrium may impair implantation through inflammatory and immune-mediated mechanisms [28,36,37]. This supports the concept that infertility in endometriosis is not exclusively ovarian but involves a broader disruption of the reproductive environment, including endometrial and systemic factors.

Therapeutic strategies in endometriosis-associated infertility remain a subject of ongoing debate. Surgical treatment may improve spontaneous conception rates in selected cases; however, its role prior to IVF remains controversial [38,39,40].

Recent meta-analyses suggest that surgical intervention, particularly in deep infiltrative endometriosis, does not consistently improve ART outcomes and should therefore be carefully individualized [41,42,43]. Moreover, excessive surgical intervention may negatively affect ovarian reserve, potentially reducing reproductive potential.

Medical management also plays a significant role, particularly in modulating the inflammatory and hormonal environment. Long-term treatment with GnRH analogues or antagonists has been explored as a strategy to improve ART outcomes, although the evidence remains heterogeneous [39,40,44]. The introduction of selective modulators such as elagolix represents a promising advancement, offering targeted suppression of estrogen-dependent pathways with potential long-term benefits [38]. Nevertheless, the optimal timing and duration of such therapies relative to ART cycles remain poorly defined.

Current clinical guidelines emphasize individualized treatment approaches based on disease phenotype, symptom severity, and reproductive goals. The ESHRE guideline on ovarian stimulation provides a structured framework for ART management, although specific recommendations for patients with endometriosis remain limited [41,42,43,44,45]. In this context, optimizing ovarian stimulation protocols while minimizing disease progression represents a key clinical challenge [42].

Importantly, emerging evidence suggests that endometriosis may coexist with other uterine pathologies, such as chronic endometritis, further complicating reproductive outcomes [25]. This coexistence underscores the need for comprehensive diagnostic strategies that integrate molecular, immunological, and clinical parameters.

Despite the growing body of evidence, significant inconsistencies remain across studies. Variability in study design, heterogeneity in patient populations, differences in disease staging, and the lack of standardized biomarkers contribute to conflicting results in the literature. Furthermore, the relative contribution of inflammatory, immune, and oxidative pathways to reproductive failure remains incompletely defined. While these mechanisms are clearly involved in disease pathophysiology, their direct causal relationship to ART outcomes remains debated.

From a clinical perspective, these findings emphasize the need for individualized patient management in endometriosis-associated infertility. Rather than applying uniform treatment strategies, clinicians should consider disease phenotype, inflammatory status, and ovarian reserve when planning ART interventions. The integration of biomarker-based approaches may further improve patient stratification and optimize therapeutic decision-making.

Beyond reproductive implications, the broader impact of endometriosis on quality of life, psychological well-being, and chronic pain should not be overlooked. Psychosocial factors and chronic pain syndromes may further exacerbate disease burden and indirectly influence reproductive outcomes [24].

Taken together, the current evidence supports a multifactorial model in which inflammatory, immune, hormonal, and structural alterations converge to impair reproductive function. Despite significant advances, important gaps remain in understanding the precise mechanisms linking these pathways to ART success.

The interpretation of these findings should be approached with caution due to several limitations inherent in the available literature. The included studies are characterized by variability in methodology, heterogeneity in patient populations, and differences in outcome definitions. Additionally, the lack of standardized biomarkers and uniform diagnostic criteria further contributes to inconsistencies across studies. The narrative nature of this review may also introduce a degree of selection bias. As a result, the relative contribution of inflammatory, immune, and oxidative mechanisms to reproductive outcomes remains incompletely defined.

Future research should focus on well-designed prospective studies and the development of standardized biomarker panels to improve the predictive accuracy and clinical applicability of current findings.

## 5. Conclusions

Endometriosis is a complex and multifactorial condition involving inflammatory, immune, hormonal, and oxidative mechanisms that may contribute to impaired reproductive function. The evidence reviewed suggests that these alterations can influence oocyte quality, embryo development, and endometrial receptivity, potentially affecting outcomes in assisted reproductive technologies (ARTs).

However, the extent of this impact remains variable across studies, likely due to differences in study design, patient populations, and disease severity. While some data indicate reduced ovarian responsiveness and cumulative live birth rates, other findings suggest that certain ART outcomes, such as implantation rates, may not be consistently affected.

Current management strategies, including surgical and medical approaches, as well as optimization of ART protocols, should therefore be carefully individualized. The available evidence does not support a single standardized approach applicable to all patients with endometriosis.

Further research is needed to better clarify the mechanisms underlying reproductive impairment and to identify reliable biomarkers that may improve patient stratification and therapeutic decision-making. Well-designed prospective studies are particularly important to validate current findings and to support the development of more targeted and personalized treatment strategies.

In conclusion, although significant progress has been made in understanding endometriosis, its impact on reproductive outcomes remains incompletely defined, highlighting the need for continued investigation and a cautious interpretation of existing data.

## Figures and Tables

**Table 2:** Immune cell alterations in endometriosis and their functional implications.

Immune Cell Type	Alteration	Mechanism	Functional Consequence	References
Macrophages	Increased activation	Cytokine and growth factor secretion	Promotes inflammation and angiogenesis	[4,5,13]
NK cells	Reduced cytotoxicity	Impaired immune surveillance	Allows survival of ectopic cells	[2,3,14]
T helper cells	Th1/Th2 imbalance	Altered cytokine profile	Sustains chronic inflammation	[4,14]
Regulatory T cells	Increased	Immunosuppressive activity	Promotes immune tolerance	[4,14]
B cells	Autoantibody production	Autoimmune activation	Contributes to systemic features	[5,13]

**Table 3:** Sources and effects of oxidative stress in endometriosis.

Source of ROS	Mechanism	Cellular Effect	Reproductive Impact	References
Activated macrophages	Inflammatory activation	Increased ROS production	Impairs oocyte environment	[3,4,19]
Ectopic endometrial tissue	Metabolic activity	Oxidative damage	Affects implantation	[3,5,20]
Peritoneal fluid alterations	Reduced antioxidants	Lipid peroxidation, DNA damage	Decreases fertility potential	[4,5,19]
Mitochondrial dysfunction	Impaired respiration	ATP depletion, apoptosis	Reduces oocyte quality	[3,19,20]

## Data Availability

No new data were created or analyzed in this study. Data sharing is not applicable to this article.
